# The Interface between Neuroscience and Neuro-Psychoanalysis: Focus on Brain Connectivity

**DOI:** 10.3389/fnhum.2016.00020

**Published:** 2016-02-03

**Authors:** Anatolia Salone, Alessandra Di Giacinto, Carlo Lai, Domenico De Berardis, Felice Iasevoli, Michele Fornaro, Luisa De Risio, Rita Santacroce, Giovanni Martinotti, Massimo Di Giannantonio

**Affiliations:** ^1^Department of Neuroscience, Imaging and Clinical Sciences and Institute for Advanced Biomedical Technologies—ITAB, University G. d’AnnunzioChieti-Pescara, Italy; ^2^Institute of Psychiatry, University G. d’AnnunzioChieti-Pescara, Italy; ^3^Dynamic and Clinical Psychology, Sapienza University of RomeRome, Italy; ^4^National Health Service (NHS), Department of Mental Health, Psychiatric Service of Diagnosis and Treatment, Hospital “G. Mazzini”Teramo, Italy; ^5^Department of Neuroscience, Reproductive Sciences and Odontostomatology, Federico II University of NaplesNaples, Italy; ^6^New York State Psychiatric Institute (NYSPI), Columbia UniversityNew York, NY, USA; ^7^Department of Neuroscience, Institute of Psychiatry and Clinical Psychology, Catholic University of the Sacred HeartRome, Italy

**Keywords:** neuropsychoanalysis, neuroscience, connectivity, schizophrenia, self, unconscious

## Abstract

Over the past 20 years, the advent of advanced techniques has significantly enhanced our knowledge on the brain. Yet, our understanding of the physiological and pathological functioning of the mind is still far from being exhaustive. Both the localizationist and the reductionist neuroscientific approaches to psychiatric disorders have proven to be largely unsatisfactory and are outdated. Accruing evidence suggests that psychoanalysis can engage the neurosciences in a productive and mutually enriching dialogue that may further our understanding of psychiatric disorders. In particular, advances in brain connectivity research have provided evidence supporting the convergence of neuroscientific findings and psychoanalysis and helped characterize the circuitry and mechanisms that underlie higher brain functions. In the present paper we discuss how knowledge on brain connectivity can impact neuropsychoanalysis, with a particular focus on schizophrenia. Brain connectivity studies in schizophrenic patients indicate complex alterations in brain functioning and circuitry, with particular emphasis on the role of cortical midline structures (CMS) and the default mode network (DMN). These networks seem to represent neural correlates of psychodynamic concepts central to the understanding of schizophrenia and of core psychopathological alterations of this disorder (i.e., ego disturbances and impaired primary process thinking).

## Introduction

Current etiological models of mental disorders are complex and multifactorial. The dichotomy between biological and psychological interpretations is outdated. The advent of non-invasive techniques to explore brain functioning has renewed interest in the interplay between biological and psychological factors, originally put forward in Freud’s (1895) “Project for a Scientific Psychology” but dismissed due to lack of sufficient scientific evidence (Panksepp, 1998; Solms and Solms-Kaplan, 2000; Solms and Turnbull, 2002; Fonagy, 2003; Schore, 2003; Solms, 2004; Mancia, 2006b).

Neuroscientific research has recently moved away from the traditional field of cognitive sciences (language, memory, attention, perception, etc.) towards the study of mental disorders, in order to investigate possible modifications in neural patterns related to clinical improvements following psychotherapeutic interventions (Kandel, 1998; Demertzi et al., 2009; Cozolino, 2010; Toyokawa et al., 2011).

Kandel (1989) was the first to highlight that learning processes can permanently modify and reinforce synaptic connections. He later focused on gene-environment interaction research and theorized a novel biological approach to psychiatry and psychotherapy, in which the latter is conceived as a learning process that can, therefore, determine changes in genes and modify the strength of synaptic connections (Kandel, 1999, 2005). Several subsequent studies have demonstrated that successful talk-based interventions may lead to significant brain changes (Beauregard, 2007). More recently, a number of studies investigated brain activation during cognitive or perceptual tasks, with the aim of identifying neural correlates of various psychopathological conditions (McGuire et al., 1994; Viinamaki et al., 1998; Dierks et al., 1999; Furmark et al., 2002; Paquette et al., 2003; Rauch, 2003; Linden, 2006; Karlsonn, 2011; Leichsenring and Rabung, 2011). The use of sophisticated neuroimaging techniques in these studies has led to a shift in the conceptualization of mental disorders, removing the emphasis on the alterations of specific brain areas and highlighting alterations in their interactions within larger neural networks.

Brain connectivity studies thus appear to be particularly promising in elucidating the complex architecture of structural and functional brain networks underlying psychological functions. Though psychiatric diagnostic categories are essentially objective and symptom-based, subjective elements (i.e., the patient’s perception of his own self and of others) are essential to a comprehensive study of mental disorders. Panksepp and Solms (2012) underscored how neuropsychoanalysis has drawn attention to the first-person experience in the neuroscientific approach to the mind, thus improving our understanding of the emotional subjective level in psychiatric disorders and promoting more patient-tailored interventions. Neuropsychoanalysis has focused on linking psychodynamic concepts to specific neuronal mechanisms. Among others, memory (Kandel, 1998; Mancia, 2004, 2006a,b), dreams (Solms, 1997, 2000; Solms and Turnbull, 2002; Mancia, 2004; Hobson, 2009; Northoff, 2011), affect (Panksepp, 1998), defense mechanisms (Fonagy, 2003; Northoff and Boeker, 2006; Feinberg, 2011; Northoff, 2011), the Self and Ego functions have been singled out as major areas of interest. Brain connectivity research has provided evidence supporting the notion that the study of the Self and Ego functions is particularly relevant to the understanding of schizophrenia; in the present paper we will, therefore, focus primarily on these two areas.

## The Neuroscience-Psychoanalysis Interface

The interconnection between psychoanalysis and neuroscience goes beyond evidence of efficacy alone. Psychoanalytic theories represent an important conceptual framework for neuroscientific findings and similarly, neuroscience may help provide the neurobiological foundations to psychoanalytic concepts.

Identifying neural correlates of specific psychodynamic mechanisms may help plan psychotherapy and other treatments for psychiatric disorders. Neuropsychoanalysis acknowledges the deep evolutionary roots of the human mind and of emotional disorders, and favors a more coherent understanding of primary-process brain affective networks (Panksepp, 2011). The psychoanalytic perspective on mental functioning can guide neuroscientific research toward a better understanding of basic psychodynamic concepts, such as the Ego, defence mechanisms, dream functions and projection of mental states (Luciani et al., 2014), as well as enhance knowledge in the fields of memory, trauma, attachment, empathy, the self. Specific topics (e.g., neural correlates of emotions and empathy) are nowadays considered important new areas of investigation (Panksepp, 1998; Gallese, 2008; Panksepp and Biven, 2012).

Theoretically, psychoanalysis anticipated the neuroscientific approach postulating the existence of hierarchical systems comprising complex mental functions resulting from interactions between interconnected brain regions. Functional neuroimaging provides a promising avenue for the study of these functional networks, though new experimental studies that consider the psychoanalytic approach to mental functioning are needed. To this end, numerous neuropsychoanalytic studies promoting a dialogue between psychoanalysis and neuroscience and investigating neuroscientific theories that can be enhanced by psychoanalytic metapsychology have been conducted (for a review, see Fotopoulou et al., 2012). In the present article, we focused on two concepts in particular, the “Self” and the “Ego functions and the unconscious”, with the ultimate goal of shedding light on specific aspects of schizophrenia. This choice was largely motivated by the fact that neuroscientific research has provided evidence supporting the notion that the study of the Self and Ego functions is particularly relevant to the understanding of schizophrenia. This implies a clinical endeavor to focus on the subjective experience of symptoms and on how perception of one’s self influences perception of the outside world. From a technical standpoint this also implies investigating the complex functioning of specific brain networks.

## Brain Connectivity

Over the last few years, several neuroimaging studies explored brain connectivity. Technically, there are three types of connectivity: (i) anatomical connectivity, defined as patterns of anatomical links between neuronal populations or anatomically defined brain regions; (ii) functional connectivity, defined as patterns of statistical correlations between distinct activated brain areas; and (iii) effective connectivity, defined as causal interactions between specific groups of neurons (Friston, 1994). The relationship between these different types of connectivity is one of the most interesting and innovative fields of experimental neuroscience. This inevitably raises the following question: “how does brain connectivity research contribute to neuropsychoanalysis?”

This new perspective provokes worthwhile questions and debate and may help further our understanding of complex mental phenomena such as consciousness, the influences of different contexts on meaning attribution, the representation of the self and of others (Molnar-Szakacs and Uddin, 2013; Ionta et al., 2014; Li et al., 2014; Rudorf and Hare, 2014; Touskova and Bob, 2015). Recent reviews (van Veluw and Chance, 2014; Murray et al., 2014) evidenced that different connectivity patterns are involved in self-representation and in the representation of others: in 193 studies healthy subjects were administered self-related tasks (Molnar-Szakacs and Uddin, 2013), while in 106 studies others-related tasks were used (Li et al., 2014). Meta-analytic connectivity modeling (van Veluw and Chance, 2014) showed selective activation of the pregenual anterior cingulate during self-related tasks and selective activation of the posterior cingulate and precuneus during others-related tasks. This meta-analysis also highlighted a shared connectivity pattern between self- and others-related tasks at the ventromedial prefrontal cortex and at the medial orbitofrontal cortex, thus providing neuroscientific evidence of the intricate relationship between representations of the self and of others. These findings may give new insight into disorders such as autism, schizophrenia and borderline personality disorder, in which the representations of the self and of others are dysfunctional or disrupted (van Veluw and Chance, 2014). It is worth noting that while modifying self-representation in relation to others is a challenging process, the interchange between self and other representations spontaneously takes place during transference-countertransference interactions in transference-focused psychotherapies as well as in other social relations. Such clinical evidence suggests that, rather than the distinct conceptualizations, the interactive dynamic relationship between self and other representations is central, (Kernberg et al., 2008; Yeomans et al., 2013). Future studies should investigate whether specific interactions between self and other representations are associated with specific brain connectivity patterns, and whether changes in these representations, as detectable in the transference dynamics, bring about modifications in brain connectivity.

## The Self

In recent years, neuroscientific work has called attention to the study of the Self, previously confined to philosophy and psychology (Gallagher, 2000; Damasio, 2003; Kircher and David, 2003). Damasio (1999, 2003) and Panksepp (1998, 2003) suggested the existence of a “proto-self” in the sensory and motor domains. A “minimal self” (Gallagher, 2000; Gallagher and Frith, 2003) or a “core or mental self” (Damasio, 1999, 2003), an “autobiographical self” (Damasio, 1999, 2003) and a “narrative self” (Gallagher, 2000; Gallagher and Frith, 2003) have also been described. Beyond differences in definitions, it is possible to identify “self-related processes”, which comprise stimuli that are experienced as strongly related to one’s own person. It is very important to consider the self-stimulus relationship: the process of relating stimuli to the self should not be considered an isolated phenomenon, but rather as embedded in a larger, more complex process that depends on the environmental context (Clark, 1999; Northoff, 2004).

The Cortical Midline Structures (CMS) are brain areas related to self-referential experiences (Kelley et al., 2002). However, specific features of the self are also related to other cerebral regions (i.e., self-agency to right posterior insula and right inferior parietal cortex, self-ownership to right parietal and ventromedial prefrontal cortex; Northoff, 2004). The self therefore results from the integration of different areas, which necessarily involves neural connectivity. Northoff (2012) suggested that CMS might represent the neural correlate of the “core self”, defined by Damasio as the continuous interaction between intero- and exteroceptive stimuli that allow to perceive the self as a unit. CMS are activated in resting state conditions, and deactivated during cognitive tasks (Gusnard and Raichle, 2001; Raichle et al., 2001). Such physiological situation is linked to the psychological condition in which the core self is replaced and masked by cognitive activity, with a subjective experience of “permanent self” that represents a permanent baseline status for other psychological activities.

According to Northoff’s theory, neuroscientific research may go beyond the function and localization-based approach, moving from the “Neural Correlates of Psychodynamics” (NCC) to the “Neural Predisposition of Psychodynamics” (NPP), which refers to the neural condition of mental contents. NPP, in terms of empirical brain function, may refer to resting state activity and its “spatiotemporal structure”, which is the result of two main characteristics of resting state activity, namely low frequency fluctuations and functional connectivity patterns. The “virtual” concept of “Spatiotemporal Structure” may be compared to the Freudian approach to the “Psychological Structure” of the psychic apparatus, in that both are related to a process and an organization rather than a physical or psychological entity. The psychological predisposition of psychodynamic processes based on the Freudian psychological structure may also be related to resting state spatiotemporal structure and the so-called neural predisposition. From this point of view, resting state activity, with its spatiotemporal features that include functional connectivity between brain regions, could represent a model associated with Freudian Ego functions and dreams (for an overview, see Northoff, 2012).

Neuroscience looked into various aspects of the Self. In studies on schizophrenia, the relationship between the Self and the other has been particularly emphasized. Since the beginning of the 19th Century, phenomenology attributed a crucial role to the self and its pre-reflective attunement to the external world in schizophrenic psychopathology (Bleuler, 1911/1950; Minkowski, 1927). More recently, schizophrenia research has focused on abnormalities of the self (so-called ego-disturbances) and schizophrenia has been primarily characterized as a disorder of the self-other relationship (Parnas et al., 2002; Kircher and David, 2003; Nelson et al., 2009). Several empirical findings suggest that altered self-experience may result in an abnormal self-other relationship (Bentall et al., 1991; Ihnen et al., 1998; Peled et al., 2000; Voss et al., 2010; Morgan et al., 2011). Aberrant neural connectivity has been proposed as a basic feature of schizophrenic pathophysiology (Volkow et al., 1988; Meyer-Lindenberg and Weinberger, 2006). Neuroimaging research suggests aberrant structural and functional connectivity in schizophrenic patients (Walterfang et al., 2006; Skudlarski et al., 2010; Woodward et al., 2011; Fornito et al., 2012). However, the links between neural connectivity and social dysfunction are still poorly understood. Ebisch et al. (2013) performed a neuroimaging study with a social perception task on first episode schizophrenic patients, with the aim of highlighting brain functioning and its correlation with prodromal symptoms and self-other distinction abilities. The results of this study showed aberrant activation in the posterior insula, with impaired self-other distinction and reduced activation in the ventral premotor cortex, which negatively correlated with self-experience disturbances. These findings portray dysfunctional social perception in schizophrenia as a complex impairment involving multiple neural processing levels (Ebisch et al., 2013). Further connectivity analyses evidenced aberrant functional interactions of the posterior insula and the ventral premotor cortex with the posterior cingulate cortex (PCC), a midline region that plays a major role in mediating self-experience, suggesting an imbalance in the processing of internally and externally guided information and its abnormal integration with self-referential processing (Ebisch et al., 2014). This study provided insights on how impairments in the self-other relationship in schizophrenia may relate to altered functional interaction patterns.

Other investigations of resting state activity and functional connectivity in schizophrenia evidenced that resting state functional connectivity within the CMS and the default mode network (DMN) tends to increase. On the other hand, functional connectivity of the Control Executive Network (CEN) is reduced in schizophrenia (Hoptman et al., 2010; Karbasforoushan and Woodward, 2012). The stronger resting state activity and functional connectivity within the CMS and the DMN could be viewed as functional correlates of a greater focus on internal mental contents that are more related to the self. Conversely, stronger resting state activity and functional connectivity within the lateral regions and the CEN are related to an increase in external mental contents and awareness (Vanhaudenhuyse et al., 2011). The opposite functioning between DMN and CEN may represent the neural correlate of internal and external mental content. The confusion between internal and external mental contents that typically characterizes schizophrenic symptoms such as thought insertion, thought withdrawal, passivity symptoms and auditory hallucinations (Sommer et al., 2012) might be related to an imbalance between the two networks. Even though the exact meaning of the resting state abnormalities for psychiatric symptoms remains unclear, Northoff (2015) suggests a direct link between abnormalities of the resting state spatiotemporal structure and psychopathological symptoms such as ego disturbances and auditory hallucinations.

Given that the CMS may well represent the neural correlate of the “core self”, it is likely that abnormal activity in the CMS underlies anomalous self-perception and self-other relationship in schizophrenia. This hypothesis warrants further investigation in studies that link specific network alterations with the distinct subjective experiences of the pathological condition. In fact, in schizophrenia altered perception of internal and external stimuli reflects qualitative disturbances in discriminating between one’s own self and others. Future research on schizophrenia should take into account neuropsychoanalytic conceptualizations and focus on the study of the “Spatiotemporal Structure”, in order to further explore the basic and subjective aspects of this disorder. In fact, psychoanalysis’ contention that the self-others relationship is central to psychological development and change suggests that basic neural mechanisms (such as CMS functioning) may be affected over time by relational experiences and the environment in general. In this view, psychoanalytic theory may provide a valuable theoretical basis for neuroscientific studies on the self and its subjective experiences.

## The Ego Functions and The Unconscious

To date, structural and functional correlates for the psychodynamic notion of unconscious processes (i.e., mental processes and contents that are defensively removed as a result of conflicting attitudes) have not been identified. In recent years, there has been an increasing interest, in unconscious processes; neuroscientific studies have, in fact, tested subliminal perceptions, implicit cognition, emotion processing and interoceptive perceptions with empirical methods (Stein et al., 2006; Craig, 2009). Though many studies indicate that unconscious processes influence awareness (for a review, see Berlin, 2011), the cognitive view of the unconscious differs from the psychodynamic notion of unconscious, which encompasses affect and motivation (Turnbull and Solms, 2007). Psychoanalytic theory holds that we are partly unaware of what we really feel. Studies on both healthy and brain-injured subjects demonstrated that even when a stimulus is not consciously perceived, it modulates neural activity and generates an emotional response. Custers and Aarts (2010) reviewed studies demonstrating the existence of an “unconscious will”, by which we persevere a goal regardless of conscious awareness.

In light of Freud’s distinction between primary and secondary processes (the functioning of the unconscious and the Ego, respectively), Carhart-Harris and Friston (2010) postulated that Freud’s description of ego functioning and development corresponds to the DMN functioning and its reciprocal exchanges with other brain networks. Freud (1895, 1900) described the secondary process as “bound” and “inhibited”, whereas the primary process is “free” and “motile”. The functional connectivity of the DMN appears to differ in childhood as compared to adulthood, and is altered in many brain disorders (Ouchi and Kikuchi, 2012). Possibly, loss of top-down control over limbic activity in hierarchically lower systems mirrors loss of ego control over the primary process. Freud held that the mode of normal waking consciousness depends on the existence of an equilibrium between the pressing forces of the primary process (entailed by the id) and the counter forces of the secondary process (entailed by the ego). This description is in line with current models of cognition based on hierarchical Bayesian inference and free energy, where backward connections from higher cortical areas work to reduce the free energy of lower areas (Carhart-Harris and Friston, 2010). From a clinical point of view, this approach provides novel insight on the pathogenesis of schizophrenia. An altered functional connectivity between limbic and cortical nodes of the DMN may predict symptoms of ego disturbance or primary process thinking in schizophrenia, mainly in the prodromal phase when these symptoms are prevalent (Parnas and Handest, 2003).

The displacement of energy from a “reservoir” to activity such as control of the external world is consistent with the functioning of the DMN and its relation with the dorsal attention system during goal-directed actions (Raichle et al., 2001). Considering that disease severity positively correlates with DMN connectivity, reduced task-evoked suppression of DMN activity observed in schizophrenia may be associated with reduced engagement with the external world (Whitfield-Gabrieli et al., 2009).

## Conclusions

A rich body of literature suggests that abnormalities in the interactions of brain network components play a vital role in psychiatric disorders, and damage to specific functional connectivity networks can result in corresponding psychopathology. Studies on brain connectivity in schizophrenia and depressive disorders have improved knowledge on how complex alterations in brain functioning and neural network interaction underlie these disorders. Both the localizationist and the reductionist neuroscientific approaches to psychiatric disorders are outdated. While structural models of mind functioning appear to be insufficient, psychoanalytic theory provides a more appropriate organizational model of the mind, that matches recent neuroscientific findings on intrinsic brain networks. Many correlations between neuroscience and the theories of psychoanalysis have, in fact, been validated, and considerable progress has been made toward identifying neural correlates of abnormal self-processing in different psychiatric disorders, as maintained by psychoanalytic conceptualizations.

Psychoanalysis anticipated the neuroscientific approach postulating the existence of hierarchical systems comprising complex mental functions resulting from interactions between interconnected brain regions. To this respect, the Freudian “Psychological Structure” appears to be consistent with the neuroscientific approach based on brain connectivity. The CMS and their networks (right posterior insula, right inferior parietal cortex, ventromedial prefrontal cortex) may represent neural correlates of the “core self”, defined as the continuous interaction between intero- and exteroceptive stimuli, allowing the self to feel as a unit (Northoff, 2012). CMS are activated in resting state condition and deactivated during cognitive tasks.

Ego functioning most likely corresponds to the activation of the DMN and its reciprocal exchanges with other brain networks. An altered functional connectivity between cortical and limbic nodes of the DMN may predict symptoms of ego disturbance, such as alterations of primary process thinking in schizophrenia or attribution of negative emotions to the self in depressive disorders.

Taken together, this evidence suggests that brain connectivity research supports the convergence of neuroscientific findings and psychoanalysis, allowing for a new understanding of some fundamental concepts and symptomatic configurations. A more in-depth understanding of the functionally relevant nodes of each network, and the interactions between them, will help us advance toward a more complete theory of self-representation in the brain. Linking these neuroscientific findings and psychoanalytic theoretical models may result in new experimental paradigms and allow for investigation of the functional changes in the brain following psychotherapy, thus ultimately improving treatments.

Though the heuristic potential of psychoanalysis may support new acquisitions on brain connectivity, there is still much to investigate to fully understand mental functioning in both physiological and pathological conditions. The progress in neuroimaging techniques and the use, besides fMRI, of other techniques such as MEG, TMS and EEG (also combined with fMRI) will surely contribute to a deeper understanding. On the other hand, dynamic brain processes underlying mental functioning are not likely to be fully understood by means of neuroimaging techniques alone. Neuroscientific studies would benefit from more appropriate experimental paradigms that emphasize subjectivity and from interpretation of data that takes into account the complexity of the human mind.

## Author Contributions

All authors listed, have made substantial, direct and intellectual contribution to the work, and approved it for publication.

## Conflict of Interest Statement

The authors declare that the research was conducted in the absence of any commercial or financial relationships that could be construed as a potential conflict of interest.
